# Mycosporine-Like Amino Acids (MAAs) in Zooplankton

**DOI:** 10.3390/md18020072

**Published:** 2020-01-23

**Authors:** Samuel Hylander

**Affiliations:** Centre for Ecology and Evolution in Microbial Model Systems—EEMiS, Linnaeus University, SE-39182 Kalmar, Sweden; Samuel.hylander@lnu.se

**Keywords:** copepod, rotifer, daphnia, cladocera, krill, photoprotective compounds, ultraviolet radiation, UV, pigments, carotenoids, database

## Abstract

Organisms have different adaptations to avoid damage from ultraviolet radiation and one such adaptation is the accumulation of mycosporine-like amino acids (MAAs). These compounds are common in aquatic taxa but a comprehensive review is lacking on their distribution and function in zooplankton. This paper shows that zooplankton MAA concentrations range from non-detectable to ~13 µg mgDW^−1^. Copepods, rotifers, and krill display a large range of concentrations, whereas cladocerans generally do not contain MAAs. The proposed mechanisms to gain MAAs are via ingestion of MAA-rich food or via symbiotic bacteria providing zooplankton with MAAs. Exposure to UV-radiation increases the concentrations in zooplankton both via increasing MAA concentrations in the phytoplankton food and due to active accumulation. Concentrations are generally low during winter and higher in summer and females seem to deposit MAAs in their eggs. The concentrations of MAAs in zooplankton tend to increase with altitude but only up to a certain altitude suggesting some limitation for the uptake. Shallow and UV-transparent systems tend to have copepods with higher concentrations of MAAs but this has only been shown in a few species. A high MAA concentration has also been shown to lead to lower UV-induced mortality and an overall increased fitness. While there is a lot of information on MAAs in zooplankton we still lack understanding of the potential costs and constraints for accumulation. There is also scarce information in some taxa such as rotifers as well as from systems in tropical, sub(polar) areas as well as in marine systems in general.

## 1. Introduction

Ultraviolet radiation constitutes a small proportion of the solar spectrum but can cause significant damage to DNA and other cellular structures and in the end affect ecosystems and human health [1,2]. UV is also a natural environmental factor that has been present throughout evolutionary time [3] but recent human-induced pollution with release of ozone depleting substances has raised concern that UV-exposure will change [1]. Organisms have different adaptations to avoid UV-induced damage for example behavioral avoidance, cell repair, antioxidant defenses and accumulation of photoprotective compounds [2,4,5,6,7,8]. Mycosporine-like amino acids, so called MAAs, are one group of such photoprotective compounds with absorption maxima ranging from 310–360 nm and they are known to occur in a wide range of both aquatic and terrestrial taxa [5,7,9]. The distribution and functions of MAAs have been covered in several general reviews [4,5,7,8] but none of these reviews have focused on the role and significance of MAAs in zooplankton. These consumers are key components in the aquatic food web transferring energy and matter from lower trophic levels, such as phytoplankton and bacteria, and are themselves a key prey items for planktivorous fish. Hence, it is important to understand the zooplankton biology and the role of MAAs in zooplankton ecology. Some of the first records of MAAs in zooplankton came from clear alpine lakes [10,11] and from marine Antarctic copepods [9], and the development of precise methods for quantifying MAAs in plankton using high-performance liquid chromatography (HPLC) was important for the development of this field [12]. The knowledge has since then expanded and this paper reviews and synthetizes the literature covering the presence and significance of MAAs in freshwater and marine zooplankton. I focus on MAA concentration ranges in different taxa of zooplankton including copepods, cladocerans, rotifers and krill in different habitats. I describe the mechanisms for gaining MAAs, seasonal dynamics as well as differences in MAA concentrations along environmental gradients. Finally, I review and discuss the function of MAAs in zooplankton.

## 2. Results and Discussion

### 2.1. Concentrations in Different Taxa

**Figure 1:**
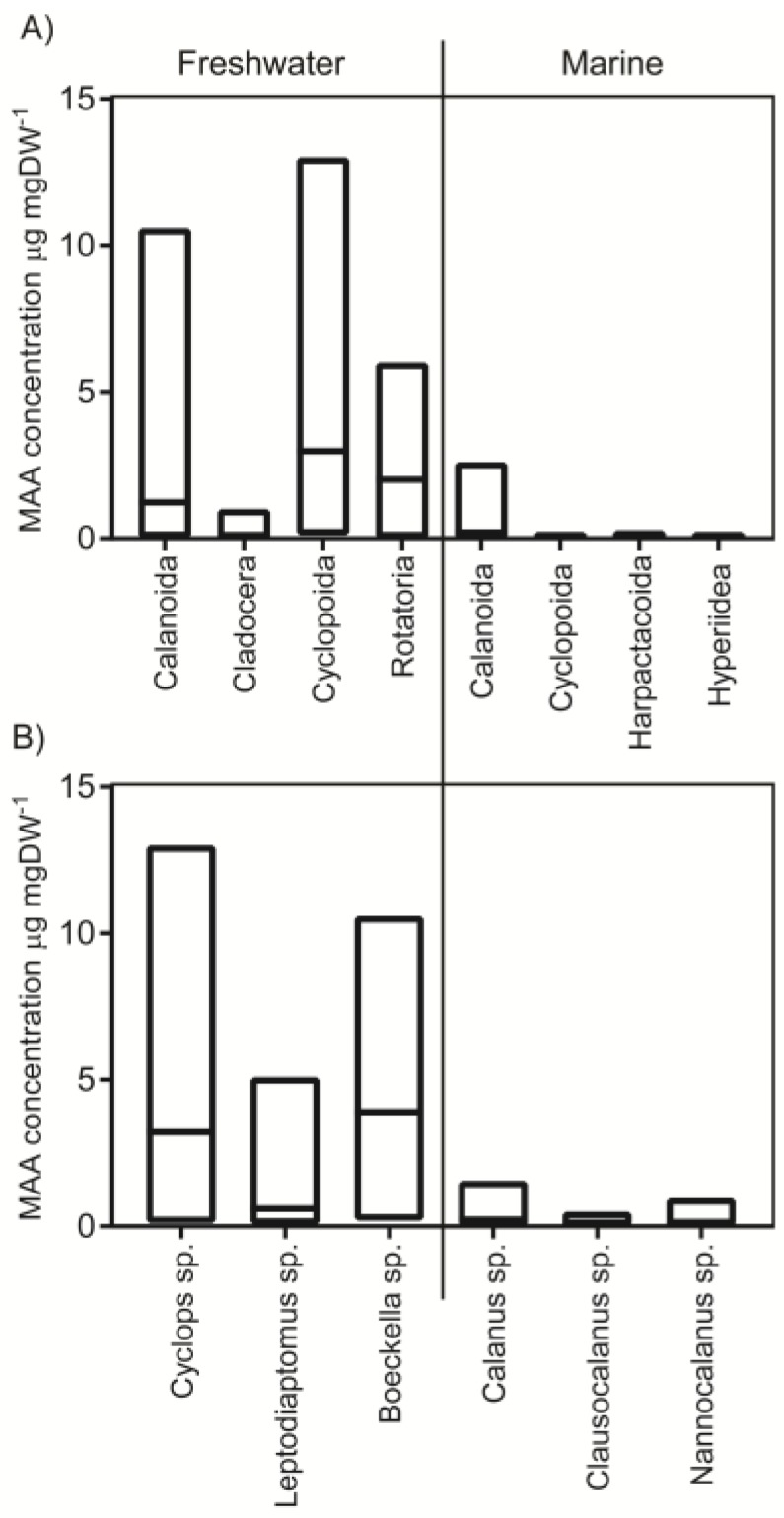
Total range of reported mycosporine-like amino acids (MAA) concentrations (box) in different taxa of zooplankton (**A**) and in the most commonly studied genera (**B**) in freshwater and marine systems. Median is illustrated by a line. All data is available in supplementary material Table S1 and has been extracted from published estimates of MAA concentrations when authors have used HPLC- methods in the quantification [14,15,16,17,18,19,20,21,22,23,24,25,26,27,28,29,30,31].

MAAs have been detected in several zooplankton taxa in a total of 43 species (Figure 1, see references in figure text). This is a small proportion of all known zooplankton species. For example, there are in the order of 13,000 copepod species worldwide and 2800 of these live in freshwater systems [13]. Concentrations in copepods generally range from close to zero up to approximately 13 µg mgDW^−1^ (i.e., concentration of MAAs per dry weight) and median concentrations in freshwater calanoid and cyclopoid copepods are 1.2 and 3.1 µg mgDW^−1^, respectively (Figure 1A). In total 16 species of calanoids and four cyclopoid species have been analyzed coming from 37 and 14 lakes, respectively. The three most common genera in the database are *Cyclops* sp., *Boeckella* sp. and *Leptodiaptomus* sp. where the two former have a larger range of reported MAA concentrations and higher median values compared to *Leoptodiaptomus* sp. (Figure 1B).

**Figure 2:**
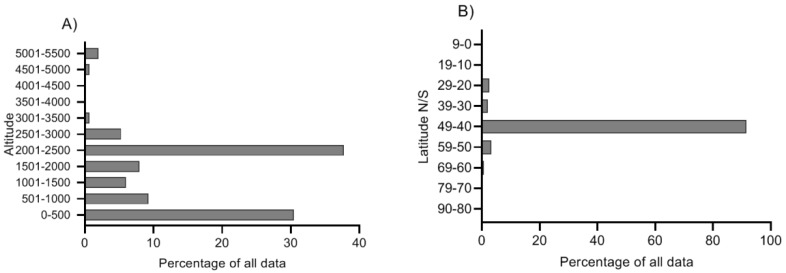
The relative distribution of MAA measurements in zooplankton in lakes at different altitudes (**A**) and latitudes (**B**; either *N* or *S*). All data are available in supplementary material Table S1 and have been extracted from published estimates of MAA concentrations where authors have used HPLC-quantification methods [14,15,16,17,18,19,20,21,22,23,24,25,26,27,28,29,30,31].

Hence, freshwater copepods are relatively well covered in the literature. However, the cyclopoid data is heavily skewed towards one species (*Cyclops abyssorum tatricus*) providing 80% of the total data on cyclopoids whereas data on calanoid copepods is more evenly distributed among species. More than 90% of the freshwater MAA estimates derive from latitudes 40–49 *N* and *S* and the data coverage around the equator and poles is scarce (Figure 2B). When it comes to altitude, most studies are performed at 0–500 m and 2000–2500 m above sea level (Figure 2A).

**Figure 3:**
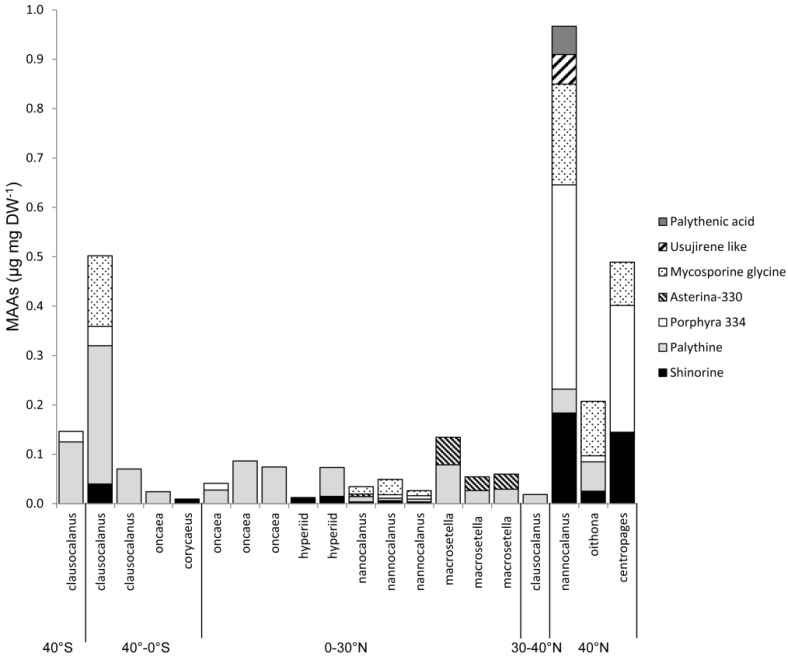
Figure from Fileman et al. (2017) illustrating the MAA concentrations in different marine copepod taxa along a transect from 40 *S* to 40 *N* [14]. Colors and patterns denote different MAAs.

Marine copepods are much less studied with data only provided from three different studies including seven species of calanoids and three cyclopoid copepod species (Figure 1, see references in figure text). Of the few species that have been analyzed they generally have smaller ranges in MAA concentrations compared to freshwater systems ranging from non-detectable to 2.6 and 0.2 µg mgDW^−1^, respectively (Figure 1; median 0.2 and 0.05 for marine calanoid and cyclopoid copepods, respectively). Even though sampling effort has been relatively low in marine zooplankton, one cruise has covered a vast transect from latitudes 40 *N* to 40 *S* (Figure 3) and another study covered several months of seasonal dynamics from the Arctic [14,19]. For example, the ecologically and economically important genus *Calanus* spp. have MAA concentrations ranging from non-detectable to 1.6 µg mgDW^−1^ but this has only been analyzed in one study [19]. For other marine species, apart from *Calanus,* only few recordings occur per genus (Figure 1 and Figure 3).

Six species of cladocerans coming from 13 different lakes have been sampled for MAAs (Figure 1 and Supplementary material Table S1). There is one example of *Daphnia* sp. containing significant concentrations of MAAs [25] but this is based on a single sample and all other analyses have found no MAAs or only in trace amounts (~0.02 µg mgDW^−1^, Figure 1). The general conclusion is therefore that cladocerans do not contain MAAs in the body tissues and trace amounts detected could be from the ingested food [24,32].

Rotifers are not as well studied as the other zooplankton groups and only five species have been analyzed from a single lake [22]. MAA concentrations range from 0–6 µg mgDW^−1^. Also *Asplanchna priodonta*, *Keratella cochlearis,* and *Polyarthra dolichoptera* have been analyzed for MAAs in another study where A. *priodonta* had no MAAs and the latter two contained a suite of different MAAs but total concentrations were not reported [32]. Krill seem to have the ability to take up MAAs from the food source [33] and in the few field data that are available concentrations ranged from 1.6–5.1 µg mgDW^−1^ (calculated to this unit assuming molecular weight of shinorine) [34]. Hence, in general MAAs seem to be present in copepods, rotifers, and krill but not in cladocerans. The number of samplings are significant when it comes to freshwater copepods but MAA concentrations have mainly been studied at latitudes 40–49 *N*/*S* and studies closer to the poles and equator are to a large extent lacking.

### 2.2. Mechanism for Gaining the MAAs (Feeding, Symbiosis, Production)

The current view is that MAAs are not produced by animals and zooplankton have to gain them in some way, either via the diet or as recently suggested via symbiotic bacteria in the gut. However, recent studies have found that some animals (e.g., some fish, amphibians, reptiles, and birds) have the pathways and ability to produce a MAA precursor called gadusol [35]. Furthermore, genes related to MAA-production have been found in sea anemones suggesting that some animals in fact may have the possibility to produce MAAs [36,37] but this has not been shown among zooplankton and the discussion below is therefore focused on dietary and symbiotic pathways for gaining MAAs. Dietary MAAs are only available in some phytoplankton and concentrations in seston vary among systems [38,39]. Several studies have analyzed if there is a correlation between field concentrations of MAAs in seston and zooplankton and this correlation is generally relatively weak to non-significant [30,32,40]. However, stronger relationships have been demonstrated if a lag phase of a couple of weeks is introduced between copepod and seston MAAs [30] suggesting that the copepod concentrations is a reflection of the previous feeding history. Furthermore, populations of the same species deriving from different lakes display different MAA dynamics suggesting that isolation of populations in areas with different UV exposure and temperatures regimes leads to local adaptation in terms of MAA accumulation capability [16].

**Figure 4:**
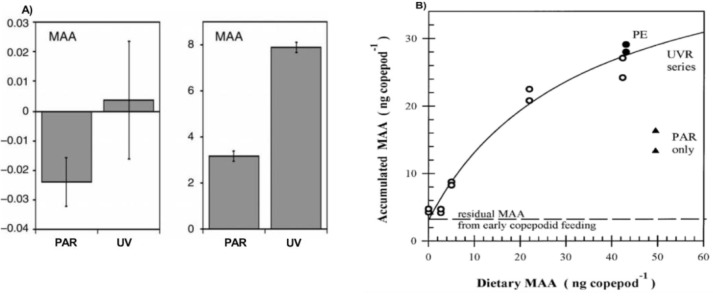
Figure adapted from Hansson et al. (2007; **A**; [17]) and Moeller et al. (2005; **B**; [21]). Left panel (**A**) illustrates the change in MAA concentrations compared to initial concentrations in two calanoid freshwater copepods, *Leptodiaptomus angustilobus* (left) and *Eudiaptomus gracilis* (right), upon laboratory UV treatment or release from UV stress (PAR—photosynthetically active radiation). Right panel (**B**) illustrates the MAA accumulation in *Leptodiaptomus minutus* at different availability of MAAs in the diet. The diet had different proportions of MAA-producing phytoplankton and copepods were raised for four weeks. For more details on methods see Hansson et al. (2007 [17]) and Moeller et al. (2005; [21]).

It has been shown several times that MAA concentrations are higher in copepods fed with food items containing MAAs as compared to controls with other food sources devoid of MAAs [21,23,40,41]. Furthermore, MAA concentrations increase even more when copepods are simultaneously exposed to UV-radiation (Figure 4A) [15,16,17,21,23,40,41,42]. This is likely because of two processes where phytoplankton exposed to UV increase their production of MAAs and this is mirrored in higher concentrations in the zooplankton [43]. This has been shown in krill (*Euphasia superba*) that increased their MAA concentrations when fed with phytoplankton earlier exposed to UV [33]. However, other experiments with a supply of replenished phytoplankton every day (not exposed to UV) suggest that zooplankton also actively accumulate MAAs during UV-exposure (Figure 4B) [21]. Field data also suggest a mechanism for active accumulation of MAAs [39]. Hence, animals exposed to UV-stress accumulate in the order of double amounts of MAAs compared to controls under the condition that MAAs are available in the food source [21].

Several examples also exist where copepods have displayed changed MAA concentrations without a food source containing MAAs [15,16,44,45]. This is suggested to be due to symbiotic bacteria in the gut providing MAAs to the zooplankton since this effect diminishes when zooplankton are exposed to antibiotic treatment [15,23]. Bacteria known to have the MAA-synthesis pathways have been found in the microbiome of calanoid copepods suggesting that this is a possible mechanism for zooplankton to gain MAAs without a food source containing these substances [23]. Symbiosis between reef building corals and microalgae can provide MAAs for the organisms [36,46] but this potential interaction has not been shown among zooplankton and MAA producing microalgae.

The mechanisms for absorption of MAAs in the zooplankton gut are not well known. Accumulation has been shown to follow a hyperbolic curve levelling off at high concentration of MAAs in the food source (Figure 4B) and has been suggested to be highly efficient even when dietary MAAs are limited [21]. Uptake rates are dependent on initial concentration with higher uptake rates in copepods with lower initial MAA concentrations [15,16]. For example, a copepod with initially relatively low concentrations of MAAs increased its concentration with 171% during an incubation [15]. When copepods do not have MAAs in the food source concentrations seem to decrease at a half-life of approximately 23 days regardless of the radiation treatment [21]. However, other studies have found that MAA concentrations in different copepod species are maintained or even increase over time when the diet lacks MAAs supposedly with help of provision of MAAs from symbiotic bacteria [15]. Finally, krill seem to be able to retain several of the initially available MAAs although exposed to several weeks of starvation [33]. Temperature also affects the MAA dynamics in copepods where MAA accumulation rates have been shown to have a linear relationship over a certain range of temperatures [15,44] and temperature dependence is population specific [16].

**Figure 5:**
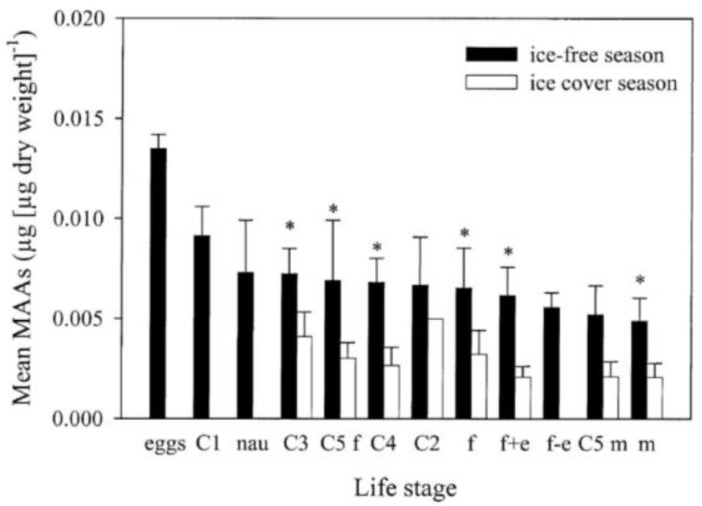
Figure adapted from Tartarotti and Sommaruga (2006; [30]) illustrating life-stage specific concentrations of MAAs in a freshwater cyclopoid copepod (*Cyclops abyssorum tatricus*) during the ice-free and ice-covered season. Life stages are eggs, nauplii, copepodite stages (C1–5), females and males.

Little is known about the fate of MAAs in the body tissues of zooplankton and how MAAs are distributed but one study has shown that concentrations generally are low in antennas, furca, and some body segments, intermediate in the cephalothorax, abdomen, some other body segments, legs, and highest in the eggs [47]. This is consistent with other studies showing that concentrations of MAAs are generally higher in eggs and nauplii compared to juveniles and adults [30,32]. This is generally interpreted as a maternal transfer of the substance since early stages of nauplii do not feed and often stay close to the surface [32]. For example, Tartarotti and Sommaruga (2006) found almost three times higher concentrations of MAAs in eggs compared to adult males [30] (Figure 5).

### 2.3. Seasonal Variation

**Figure 6:**
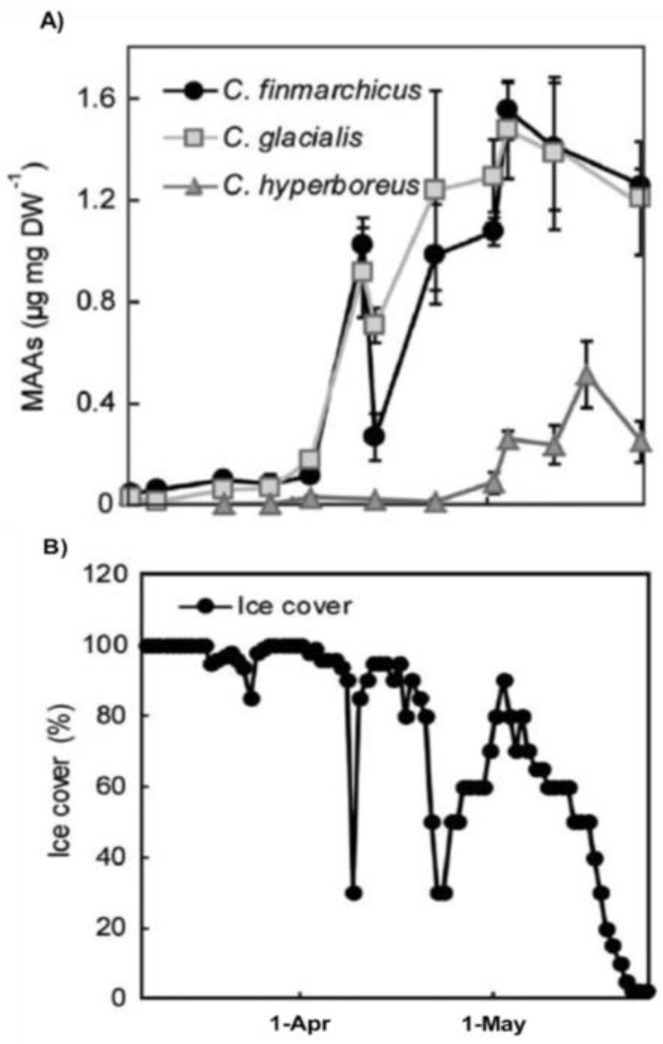
Figure adapted from Hylander et al. (2015; [19]) illustrating the concentrations of MAAs (**A**) in three marine calanoid copepods (C. *finmarchicus*, C. *glacialis* and C. *hyperboreus*) from 8 March to 24 May during an Arctic spring. Panel (**B)** shows ice cover in percent over the same time period.

Seasonal variation in copepod MAA concentrations has been studied in one marine and a few freshwater systems [19,21,24,27,30]. MAAs are usually detected throughout the year but concentrations are generally low in winter [19,27,30]. As spring progresses and ice disappears, there is generally an increase followed by a peak during spring- summer followed by a reduction in concentrations during fall [19,21,24,27,30]. This has been studied in more detail in three species of marine *Calanus* spp. And one species of freshwater *Cyclops* [19,30]. *Calanus* spp. over-winter at great depths and emerge to surface waters during spring for feeding and reproduction but are at this time exposed to the risk of UV-damage (Figure 6).

MAA concentrations increase rapidly in C. *finmarchicus* and C. *glacialis* during the Arctic ice-out and this is also a period when chlorophyll concentrations increased rapidly in the system [19]. Another species, C. *hyperboreus*, has lower concentrations of MAAs during ice-out but eventually concentrations increase as spring progresses [19] (Figure 6). This could be attributed to different life-histories among *Calanus* species where this latter species migrated to surface waters later than C. *finmarchicus* and C. *glacialis* [19]. Freshwater copepod MAA seasonal dynamics has been studied during an entire year and concentrations increased rapidly after ice-out and were generally highest during the ice-free period, peaking during summer [30]. Concentrations then decrease during autumn and had lowest values in March and April before ice-out in late May-early June [30]. Concentrations were on average approximately three times higher during the ice-free period than during ice conditions and the seasonal patterns were relatively similar among different life stages [30] (Figure 5). Seasonal dynamics in krill is available in one study suggesting no particular differences under seasonal changes in UV levels and MAA-diet supply [34].

### 2.4. Variation along Different Environmental Gradients

**Figure 7:**
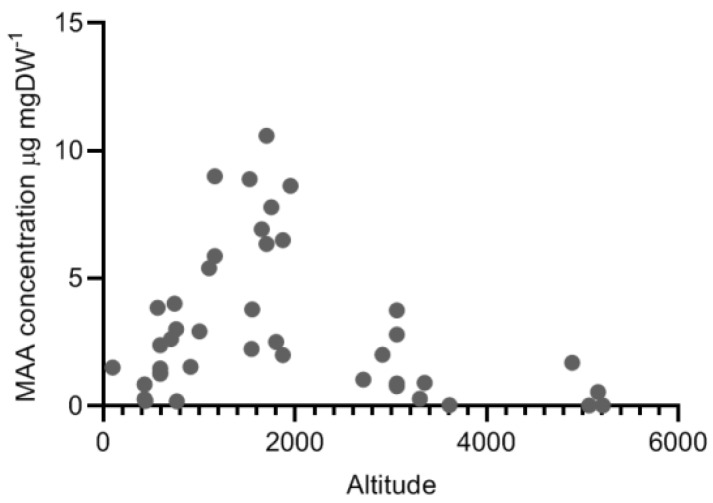
Range of MAA concentrations at different altitudes (m above sea level) in the most commonly occurring group of copepods in the MAA-literature, i.e., freshwater calanoid copepods. Each data point shows the MAA concentration per species in each lake (some lakes have several species of calanoid copepods). The average concentration for the ice-free period is shown herewhen several measurements of the same species were available from the same lake. Also see Figure 1 and data in Supplementary Material Table S1.

Much attention in the literature has been devoted to increase our understanding of how MAA concentrations in zooplankton vary along different environmental gradients. Copepods within the same species tend to have higher MAA concentrations in lakes at higher altitudes [16] and significant linear relationships within species between MAAs and altitude have been demonstrated several times [28,32]. Comparing all calanoid species available in the present data set (Supplementary Material Table S1, Figure 1) there seems to be a positive linear relationship up to approximately 2000 m above sea level (Figure 7). However, at higher altitudes concentrations then tend to be lower. For example, copepods sampled in lakes in the Himalayas (4800–5200 m above sea level) had MAA concentrations ranging from 0.03-1.7 µg mgDW^−1^ which is in the lower range of reported MAA concentrations [26] (Figure 1 for comparison). Considering other environmental gradients there are also several examples of significant relationships between MAAs and temperature as well as UV transparency [31], which is consistent with the fact that high altitude lakes generally are cold and usually have a high UV-transparency.

**Figure 8:**
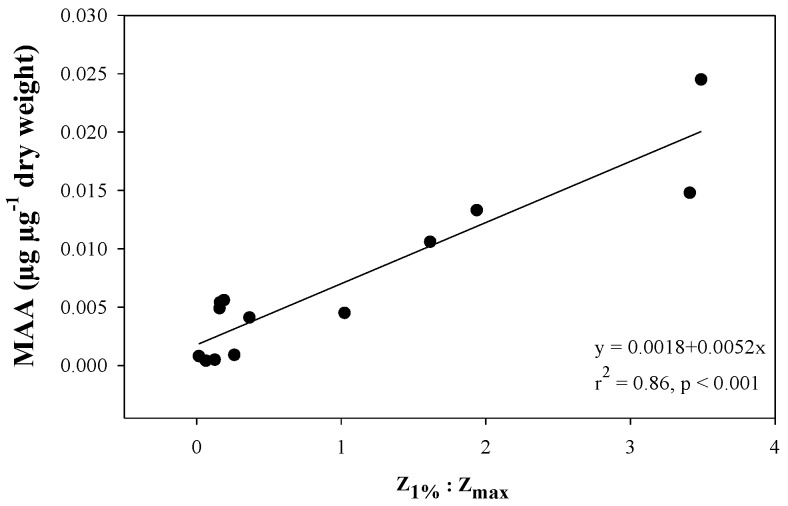
Adapted from Tartarotti et al. (2001; [32]. MAA concentrations in a freshwater cyclopoid copepod along a gradient of the ratio of 1% attenuation depth (320 nm; Z1%) and the maximum lake depth (Zmax).

An extension of this reasoning is the relationship found between MAA concentrations in zooplankton and the ratio between the 1% attenuation depth of UV and the maximum depth put forward by Tartarotti et al. (2001) [32] (Figure 8). Such relationships were also demonstrated in another study in three out of four copepod taxa but variability was relatively high suggesting that other factors may be of importance [24]. The rationale for using such a relationship is to understand if deep transparent lakes have a depth refuge where zooplankton can avoid UV-exposure. Continuing with turbidity one study has found that lakes with high turbidity have zooplankton with relatively low MAA concentrations [48], and a survey of six lakes along a transparency gradient found that copepods in the clear lakes had in the order of 11 times more MAAs compared to copepods deriving from glacier-fed turbid lakes [31].

Comparing MAA concentrations between freshwater and marine systems is difficult since few measurements exist from marine habitats and lake data is skewed towards high altitude systems. With the few comparable data that exists (low altitudes and similar latitude) it seems like concentrations in marine and freshwater systems are approximately the same [41]. Furthermore, MAA concentrations tend to be higher in copepods residing close to the surface or at mid-depths compared to deeper down as has been shown in both marine and freshwater systems [30,42]. Rotifer species with relatively high MAA concentrations have also been shown to have a surface dwelling life style compared to other species that generally reside at deeper depths [22].

Finally, some studies suggest that there is a cost associated with high concentrations of MAAs in zooplankton in terms of higher visibility and predation. For example, surface dwelling zooplankton have been shown to be less UV transparent compared to deeper dwelling zooplankton [49] and fish foraging is elevated in the presence of UV compared to non-UV treatments [50]. However, experimental exposure to fish cues did not have any effect on MAA concentrations in freshwater copepods [40].

### 2.5. Function of MAAs in Zooplankton

As summarized here, zooplankton tend to have high concentrations of MAAs when residing in habitats where UV-exposure is high. Examples of these systems are shallow and clear high altitude lakes and during periods when marine and freshwater zooplankton are migrating to the surface for feeding. It was early hypothesized that MAAs provide zooplankton with UV-screening since this had been demonstrated in other types of organisms [10,11]. One of the main results supporting this hypothesis is that zooplankton exposed to UV radiation accumulate more MAAs compared to individuals only exposed to visible light (see *Mechanism for gaining the MAAs*). Furthermore, field studies find significant relationships between MAA concentrations and different metrics for UV-threat [24,32,40].

**Figure 9:**
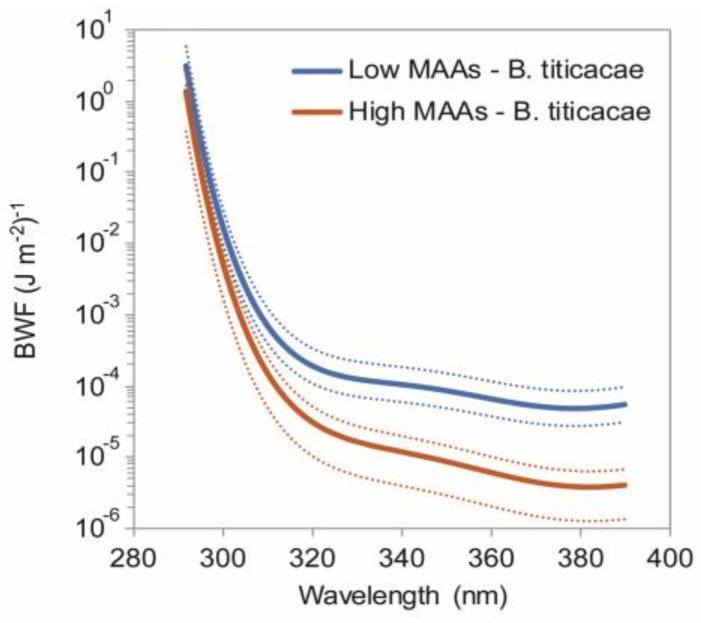
Biological weighting functions (BWF) of the copepod B. *titicacae* containing either high or low concentrations of MAAs [51]. Raw data for this figure was kindly provided from by W. Helbling.

Helbling et al. (2002) observed that UV-induced mortality was higher in individuals from the same species if they had low concentrations of MAAs [51]. Other studies have also found UV-induced mortality to be higher in populations of the same species with lower concentrations of MAAs [21,52]. Comparing different species it has also been suggested that species having large amounts of MAAs are more tolerant to UV-exposure compared to other species without this photoprotection [53]. A biological weighting function was developed to assess the effects of different wavelengths of UV on copepod mortality (Figure 9). This analysis shows that MAAs seem to provide UV-protection in the UVA and in parts of the UVB spectrum [51].

Exposure to UV radiation leads to DNA damage [54] and a study comparing four different populations of copepods found similar DNA damage in all populations upon UV-exposure but background levels were lower in the population with the lowest MAA concentration (and antioxidant capacity) leading to an overall higher total DNA damage among copepods from this population [29]. The molecular mechanisms are now being examined and the expression of genes associated with stress have been shown to be modulated by the photoprotection level (including MAAs) in the animal [27].

**Figure 10:**
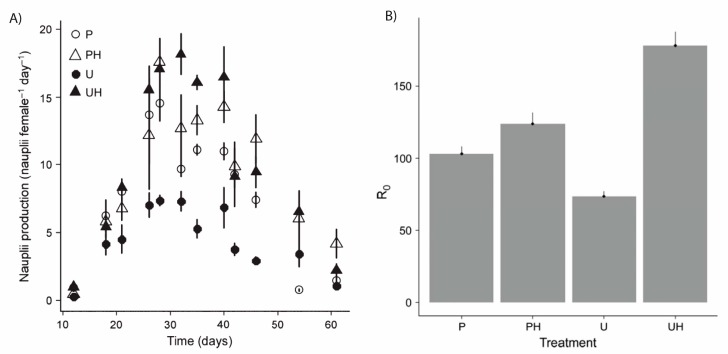
Adapted from Hylander et al. 2014 [41]. (**A**) Nauplii production in a cohort of *Acartia tonsa* raised from eggs to death. P = only exposed to PAR; U = exposed to UV-radiation, PH = PAR and MAA rich food, UH = UV exposure and MAA rich food. Subpanel. (**B**) The net reproductive rate (*R*_0_ for this cohort of *Acartia tonsa*). *R*_0_ is a proxy for fitness.

Moving beyond mortality some studies now show that MAAs (together with other photoprotective strategies) enable zooplankton to maintain feeding upon UV exposure [55]. It has also been shown that reproduction in copepods is reduced when MAAs are not available in the food source, but only under the condition that other UV-defenses are simultaneously at a low level [40]. For example, freshwater copepods reduced their carotenoid concentration upon exposure of fish cues and this led to reduced nauplii production if MAA were not available in the food source [40]. To assess if MAAs provide overall higher fitness to zooplankton one has to consider the entire life-time reproductive success. This has been done in one study where a cohort of a marine copepod was studied from the egg stage to death under UV-exposure and at varying availability of MAAs in the food source [41] (Figure 10). Several fitness parameters including somatic growth and egg quality were negatively affected by UV exposure especially when MAAs were not available in the food source [41] (Figure 10). Hence, exposure to UV radiation without availability of MAAs in the food source reduced the net reproductive rate, i.e., the average number of offspring that an individual delivers to the next generation (a commonly used proxy for fitness) [41].

It is now widely accepted that MAAs provide photoprotection to a range of aquatic taxa [4,5,7,8,56] but it is also important to take into account other strategies that organisms use to avoid damage from UV. These other adaptations can co-vary with MAA concentrations, for example behavioral responses, accumulation of carotenoids, and anti-oxidant enzymes [4]. For example, low concentrations of MAAs and absence of other photoprotective strategies such as accumulation of carotenoids or avoidance [4] have been suggested to be compensated by higher antioxidant defenses [57,58,59]. Furthermore, *Daphnia* has shown to behaviorally avoid UV radiation by migrating to deeper depths (e.g., [17,60]). Copepods show a more diverse behavioral response to UV with some examples of avoidance, whereas some species display little response or even attraction (e.g., [20,61,62,63]). Differences in behavioral avoidance in response to UV have been suggested to be due to higher amounts of photoprotective compounds in copepods compared to cladocerans allowing them to utilize surface waters without being harmed by UV [17,64]. In all, organisms have to optimize the UV defenses without spending too much energy in the process, to enable them to utilize habitats that are dangerous in terms of UV exposure [17].

Finally, some data have also emerged suggesting that MAAs can be beneficial for zooplankton for other reasons than as sunscreens. For example, studies have shown that MAA concentrations can increase under dark conditions at low temperatures [16] and another study demonstrated stimulated fitness under UV exposure when MAAs were abundant as compared to PAR treatments [41]. It has been suggested that MAAs should be regarded as multi-purpose substances potentially involved in osmoregulation, nitrogen storage, and antioxidant systems [5,16,41] but these hypotheses have not been explicitly tested among zooplankton. Antioxidant capacity of mycosporine-glycine with degradation of harmful reactive oxygen species has been demonstrated in several model systems [65,66,67]. This suggests that at least some of the MAAs provide aquatic organisms with antioxidant capacity [5,66].

## 3. Gaps of Knowledge

Research on MAAs in zooplankton has been very active in the last 30 years providing a good coverage of the mechanisms for accumulation and field patterns. However, the research is heavily skewed toward latitudes 40–50 *N*/*S* and little information is available from other latitudes including the tropics and polar regions. There are also relatively few estimates available from marine systems. Future research needs to disentangle when MAA accumulation is constrained as suggested in high altitude lakes and we need better understanding of other potential benefits of MAAs in terms of nitrogen storage as well as MAAs in antioxidant and osmoregulation systems. MAA provision to zooplankton by gut microbiota has been suggested but it needs to be shown if this is a wide-spread phenomenon in many species and taxa. It would also be beneficial if the significance of MAAs was possible to quantify without co-variation with other UV-protective adaptations such as antioxidant defenses, pigmentation, and avoidance.

## 4. Materials and Methods

Literature was collected using Web of Science Core collection (4th of September 2019) with the search strings: mycospor* AND zooplankton OR mycospor* AND copepod (51 articles found). An additional database search was performed using the Scopus database (14 Jan 2020) to confirm that the initial literature coverage was comprehensive. Using search strings mycospor* AND zooplankton OR mycospor* AND copepod (limiting the search to articles in English with the keyword “Zooplankton”; 89 articles found, most of them overlapping with the initial literature search). All of these articles together with their reference lists were used to compile the present synthesis (final number of references used was 67). From this literature, all published data on MAA concentrations in zooplankton were also retrieved (see Supplementary Material Table S1, in total 18 references) including rotifers, cladocerans, copepods, and krill, and data were extracted from tables and from figures (mean values) using WebPlotDigitilizer (https://apps.automeris.io/wpd/). Each estimate of MAA concentration per lake, species, and time point was included in the data extraction. Hence, for some species there is data from the same species from the same lake at different times of the year. The criteria for including concentration data were: Had to include field concentrations (i.e., not after experimental treatments); were analyzed with HPLC-methods; and that taxonomic information was available (a least at genus level). I also extracted background data from the same articles on system (freshwater or marine systems), altitude, latitude, UVB transparency of the system (1% attenuation depth, 320 nm), maximum depth, sampling time, and site. This data together with the literature was used to illustrate the patterns and ranges of MAA concentrations in different systems and along environmental gradients focusing on the distribution and function of MAAs in zooplankton.

## Supplementary Materials

The following are available online at https://www.mdpi.com/1660-3397/18/2/72/s1, Table S1. Database MAAs in zooplankton.
