# Supplementary material for: Mycosporine-Like Amino Acids (MAAs) in Zooplankton
Source: Mar Drugs. 2020 Jan 23;18(2):72. doi: 10.3390/md18020072 (PMC7073964; doi:10.3390/md18020072)
Supplement: Supplementary file 1 [file marinedrugs-18-00072-s001.pdf]

Supplementary material - Samuel Hylander Title: Mycosporine-like amino acids (MAAs) in zooplankton

S1: Database of MAA concentrations in zooplankton. All concentrations have been recalculated to the unit µg mgDW^{-1} (i.e. µg total MAA concentration per mg dry weight). For data selection and extraction see material and methods.
Each estimate available in the literature of MAA concentration per lake, species and time point was included in the data extraction. Hence, for some species there is data from the same species from the same lake at different parts of the year.
The database also contains background data collected from the same articles including type of system (marine/freshwater), altitude, latitude, UVB transparency, maximum depth, month and year of sampling, location, zooplankton life stage and type of MAA (key to the right of the table).
N/A = not available, N/S = north or south, Maximum depth of lake = Zmax
Color coding of the rows indicates the different references used alternating between blue and green

Taxa	Species	Total MAA concentration (µg mgDW^{-1})	Reference	Freswater/Marine	Altitude (m, a s l)	Latitude (°)	N/S	UVB transparency (m, 1% at 320 nm)	Zmax (m)	Month	Year	System	Zooplankton life stage	Type of MAA detected (key to the right)	Other comments
Calanoid copepod	Centropages sp.	0.49	Fileman et al. 2017	Marine	0	40	N	N/A	N/A	Oct - Nov	2010	Atlantic		SH, PO, MY	
Calanoid copepod	Clausocalanus sp	0.15	Fileman et al. 2017	Marine	0	40	S	N/A	N/A	Oct - Nov	2010	Atlantic		PA, PO	
Calanoid copepod	Clausocalanus sp	0.50	Fileman et al. 2017	Marine	0	40-0	S	N/A	N/A	Oct - Nov	2010	Atlantic		SH,PA, PO, MY	
Calanoid copepod	Clausocalanus sp	0.07	Fileman et al. 2017	Marine	0	40-0	S	N/A	N/A	Oct - Nov	2010	Atlantic		PA	
Calanoid copepod	Clausocalanus sp	0.02	Fileman et al. 2017	Marine	0	30-40	N	N/A	N/A	Oct - Nov	2010	Atlantic		PA	
Calanoid copepod	Nannocalanus sp.	0.03	Fileman et al. 2017	Marine	0	0-30	N	N/A	N/A	Oct - Nov	2010	Atlantic		SH, PA, MY et al.	Type of MAA not visible in figure
Calanoid copepod	Nannocalanus sp.	0.05	Fileman et al. 2017	Marine	0	0-30	N	N/A	N/A	Oct - Nov	2010	Atlantic		SH, PA, MY et al.	Type of MAA not visible in figure
Calanoid copepod	Nannocalanus sp.	0.03	Fileman et al. 2017	Marine	0	0-30	N	N/A	N/A	Oct - Nov	2010	Atlantic		SH, PA, MY et al.	Type of MAA not visible in figure
Calanoid copepod	Nannocalanus sp.	0.97	Fileman et al. 2017	Marine	0	40	N	N/A	N/A	Oct - Nov	2010	Atlantic		SH, PA, PO, MY, US, P	
Cyclopoid copepod	Corycaeus sp.	0.01	Fileman et al. 2017	Marine	0	40-0	S	N/A	N/A	Oct - Nov	2010	Atlantic		SH	
Cyclopoid copepod	Oithona sp.	0.21	Fileman et al. 2017	Marine	0	40	N	N/A	N/A	Oct - Nov	2010	Atlantic		SH, PA, PO, MY	
Cyclopoid copepod	Oncaea sp.	0.02	Fileman et al. 2017	Marine	0	40-0	S	N/A	N/A	Oct - Nov	2010	Atlantic		PA	
Cyclopoid copepod	Oncaea sp.	0.04	Fileman et al. 2017	Marine	0	0-30	N	N/A	N/A	Oct - Nov	2010	Atlantic		PA, PO	
Cyclopoid copepod	Oncaea sp.	0.09	Fileman et al. 2017	Marine	0	0-30	N	N/A	N/A	Oct - Nov	2010	Atlantic		PA	
Cyclopoid copepod	Oncaea sp.	0.07	Fileman et al. 2017	Marine	0	0-30	N	N/A	N/A	Oct - Nov	2010	Atlantic		PA	
Harpacticoida	Macrosetella sp.	0.14	Fileman et al. 2017	Marine	0	0-30	N	N/A	N/A	Oct - Nov	2010	Atlantic		PA, AS	
Harpacticoida	Macrosetella sp.	0.06	Fileman et al. 2017	Marine	0	0-30	N	N/A	N/A	Oct - Nov	2010	Atlantic		PA, AS	
Harpacticoida	Macrosetella sp.	0.06	Fileman et al. 2017	Marine	0	0-30	N	N/A	N/A	Oct - Nov	2010	Atlantic		PA, AS	
Hyperiidea	Hyperiid sp.	0.01	Fileman et al. 2017	Marine	0	0-30	N	N/A	N/A	Oct - Nov	2010	Atlantic		SH	
Hyperiidea	Hyperiid sp.	0.07	Fileman et al. 2017	Marine	0	0-30	N	N/A	N/A	Oct - Nov	2010	Atlantic		SH, PA	
Calanoid copepod	Boeckella antiqua	1.00	García et al. 2010	Freswater	907	41	S	N/A	1.5	Sept-Jan	2007-2008	Los Juncos		SH, PO, MY, MAA332	
Calanoid copepod	Boeckella gracilipes	3.02	García et al. 2014	Freswater	758	41	S	0.84	12	Dec	2009	Morenito		SH, PO, MAA332	Unit of total MAAs according to text in result section
Calanoid copepod	Boeckella gracilipes	8.90	García et al. 2014	Freswater	1525	41	S	2.07	5	Nov	2009	Verde		PO, MY, P, MAA332	Unit of total MAAs according to text in result section
Calanoid copepod	Eudiaptomus gracilis	0.45	Hansson et al. 2007	Freswater	95	55	N	1.56	10	Oct	2005	Dalby		N/A	
Calanoid copepod	Leptodiaptomus angustilobus	0.05	Hansson et al. 2007	Freswater	N/A	67	N	N/A	N/A	Aug	2005	Lakes 10 and 11 in Kolyusjin Bay		N/A	
Calanoid copepod	Acartia sp.	2.60	Hylander and Jephson 2010	Marine	0	55	N	N/A	N/A	April	2007	Öresund		N/A	Data on type of MAA available but only after experimental incubation
Calanoid copepod	Eudiaptomus gracilis	3.00	Hylander et al. 2009b	Freshwater	95	55	N	1.6	N/A	May	2006	Dalby		N/A	
Calanoid copepod	Calanus finmarchicus	0.05	Hylander et al. 2015	Marine	0	69	N	4.8	N/A	March	2012	Disco Bay, Greenland		N/A	
Calanoid copepod	Calanus finmarchicus	0.06	Hylander et al. 2015	Marine	0	69	N	4.8	N/A	March	2012	Disco Bay, Greenland		N/A	
Calanoid copepod	Calanus finmarchicus	0.11	Hylander et al. 2015	Marine	0	69	N	4.8	N/A	March	2012	Disco Bay, Greenland		N/A	
Calanoid copepod	Calanus finmarchicus	0.09	Hylander et al. 2015	Marine	0	69	N	4.8	N/A	March	2012	Disco Bay, Greenland		N/A	
Calanoid copepod	Calanus finmarchicus	0.11	Hylander et al. 2015	Marine	0	69	N	4.8	N/A	April	2012	Disco Bay, Greenland		N/A	
Calanoid copepod	Calanus finmarchicus	1.03	Hylander et al. 2015	Marine	0	69	N	4.8	N/A	April	2012	Disco Bay, Greenland		N/A	
Calanoid copepod	Calanus finmarchicus	0.27	Hylander et al. 2015	Marine	0	69	N	3.3	N/A	April	2012	Disco Bay, Greenland		N/A	
Calanoid copepod	Calanus finmarchicus	0.99	Hylander et al. 2015	Marine	0	69	N	3.3	N/A	April	2012	Disco Bay, Greenland		N/A	
Calanoid copepod	Calanus finmarchicus	1.08	Hylander et al. 2015	Marine	0	69	N	3.3	N/A	May	2012	Disco Bay, Greenland		N/A	
Calanoid copepod	Calanus finmarchicus	1.56	Hylander et al. 2015	Marine	0	69	N	3.3	N/A	May	2012	Disco Bay, Greenland		N/A	
Calanoid copepod	Calanus finmarchicus	1.42	Hylander et al. 2015	Marine	0	69	N	3.3	N/A	May	2012	Disco Bay, Greenland		N/A	
Calanoid copepod	Calanus finmarchicus	1.26	Hylander et al. 2015	Marine	0	69	N	3.3	N/A	May	2012	Disco Bay, Greenland		N/A	
Calanoid copepod	Calanus glacialis	0.03	Hylander et al. 2015	Marine	0	69	N	4.8	N/A	March	2012	Disco Bay, Greenland		N/A	
Calanoid copepod	Calanus glacialis	0.01	Hylander et al. 2015	Marine	0	69	N	4.8	N/A	March	2012	Disco Bay, Greenland		N/A	
Calanoid copepod	Calanus glacialis	0.06	Hylander et al. 2015	Marine	0	69	N	4.8	N/A	March	2012	Disco Bay, Greenland		N/A	
Calanoid copepod	Calanus glacialis	0.07	Hylander et al. 2015	Marine	0	69	N	4.8	N/A	March	2012	Disco Bay, Greenland		N/A	
Calanoid copepod	Calanus glacialis	0.18	Hylander et al. 2015	Marine	0	69	N	4.8	N/A	April	2012	Disco Bay, Greenland		N/A	
Calanoid copepod	Calanus glacialis	0.92	Hylander et al. 2015	Marine	0	69	N	4.8	N/A	April	2012	Disco Bay, Greenland		N/A	
Calanoid copepod	Calanus glacialis	0.71	Hylander et al. 2015	Marine	0	69	N	3.3	N/A	April	2012	Disco Bay, Greenland		N/A	
Calanoid copepod	Calanus glacialis	1.24	Hylander et al. 2015	Marine	0	69	N	3.3	N/A	April	2012	Disco Bay, Greenland		N/A	
Calanoid copepod	Calanus glacialis	1.30	Hylander et al. 2015	Marine	0	69	N	3.3	N/A	May	2012	Disco Bay, Greenland		N/A	
Calanoid copepod	Calanus glacialis	1.48	Hylander et al. 2015	Marine	0	69	N	3.3	N/A	May	2012	Disco Bay, Greenland		N/A	
Calanoid copepod	Calanus glacialis	1.39	Hylander et al. 2015	Marine	0	69	N	3.3	N/A	May	2012	Disco Bay, Greenland		N/A	
Calanoid copepod	Calanus glacialis	1.21	Hylander et al. 2015	Marine	0	69	N	3.3	N/A	May	2012	Disco Bay, Greenland		N/A	
Calanoid copepod	Calanus hyperboreus	0.00	Hylander et al. 2015	Marine	0	69	N	4.8	N/A	March	2012	Disco Bay, Greenland		N/A	
Calanoid copepod	Calanus hyperboreus	0.00	Hylander et al. 2015	Marine	0	69	N	4.8	N/A	March	2012	Disco Bay, Greenland		N/A	
Calanoid copepod	Calanus hyperboreus	0.01	Hylander et al. 2015	Marine	0	69	N	4.8	N/A	April	2012	Disco Bay, Greenland		N/A	
Calanoid copepod	Calanus hyperboreus	0.00	Hylander et al. 2015	Marine	0	69	N	4.8	N/A	April	2012	Disco Bay, Greenland		N/A	
Calanoid copepod	Calanus hyperboreus	0.00	Hylander et al. 2015	Marine	0	69	N	3.3	N/A	April	2012	Disco Bay, Greenland		N/A	
Calanoid copepod	Calanus hyperboreus	0.07	Hylander et al. 2015	Marine	0	69	N	3.3	N/A	April	2012	Disco Bay, Greenland		N/A	
Calanoid copepod	Calanus hyperboreus	0.25	Hylander et al. 2015	Marine	0	69	N	3.3	N/A	May	2012	Disco Bay, Greenland		N/A	
Calanoid copepod	Calanus hyperboreus	0.22	Hylander et al. 2015	Marine	0	69	N	3.3	N/A	May	2012	Disco Bay, Greenland		N/A	
Calanoid copepod	Calanus hyperboreus	0.50	Hylander et al. 2015	Marine	0	69	N	3.3	N/A	May	2012	Disco Bay, Greenland		N/A	
Calanoid copepod	Calanus hyperboreus	0.23	Hylander et al. 2015	Marine	0	69	N	3.3	N/A	May	2012	Disco Bay, Greenland		N/A	
Calanoid copepod	Leptodiaptomus minutus	0.45	Moeller et al. 2015	Freshwater	428	41	N	N/A	24	April	2002	Lake Giles		N/A	
Calanoid copepod	Leptodiaptomus minutus	0.59	Moeller et al. 2015	Freshwater	428	41	N	N/A	24	May	2002	Lake Giles		N/A	
Calanoid copepod	Leptodiaptomus minutus	0.60	Moeller et al. 2015	Freshwater	428	41	N	N/A	24	June	2002	Lake Giles		N/A	
Calanoid copepod	Leptodiaptomus minutus	1.69	Moeller et al. 2015	Freshwater	428	41	N	N/A	24	July	2002	Lake Giles		N/A	
Calanoid copepod	Leptodiaptomus minutus	1.23	Moeller et al. 2015	Freshwater	428	41	N	N/A	24	Aug	2002	Lake Giles		N/A	
Calanoid copepod	Leptodiaptomus minutus	1.03	Moeller et al. 2015	Freshwater	428	41	N	N/A	24	Sept	2002	Lake Giles		N/A	
Calanoid copepod	Leptodiaptomus minutus	0.50	Moeller et al. 2015	Freshwater	428	41	N	N/A	24	Oct	2002	Lake Giles		N/A	
Calanoid copepod	Leptodiaptomus minutus	0.52	Moeller et al. 2015	Freshwater	428	41	N	N/A	24	Nov	2002	Lake Giles		N/A	
Calanoid copepod	Leptodiaptomus minutus	2.49	Moeller et al. 2015	Freshwater	428	41	N	N/A	24	Sept	2001	Lake Giles		N/A	
Calanoid copepod	Leptodiaptomus minutus	0.70	Moeller et al. 2015	Freshwater	428	41	N	N/A	24	Oct	2001	Lake Giles		N/A	
Calanoid copepod	Leptodiaptomus minutus	0.89	Moeller et al. 2015	Freshwater	428	41	N	N/A	24	Dec	2001	Lake Giles		N/A	
Rotifer	Keratella cochlearis	0.00	Obertegger et al. 2008	Freshwater	1178	46	N	3.671328671	39	Aug	2003	Lake Tovel		PA	UV transparency extracted from figure
Rotifer	Polyarthra dolichoptera	6.00	Obertegger et al. 2008	Freshwater	1178	46	N	3.671328671	39	July-Aug	2003 and 2005	Lake Tovel		SH, PO, PA, AS, MG	UV transparency extracted from figure
Rotifer	Polyarthra pectinata	2.00	Obertegger et al. 2008	Freshwater	1178	46	N	3.671328671	39	Aug	2003	Lake Tovel		PO, PA, AS	UV transparency extracted from figure
Rotifer	Syncaeta kitina	0.00	Obertegger et al. 2008	Freshwater	1178	46	N	3.671328671	39	Aug	2003	Lake Tovel		Not detected	UV transparency extracted from figure
Rotifer	Synchaeta grandis	2.00	Obertegger et al. 2008	Freshwater	1178	46	N	3.671328671	39	Aug	2003	Lake Tovel		PO, PA, AS	UV transparency extracted from figure
Calanoid copepod	Boeckella antiqua	2.08	Perez et al. 2012	Freshwater	907	41	S	N/A	1.5	N/A	N/A	Los Juncos		N/A	Zmax from Garcia et al. 2010
Calanoid copepod	Anglaodiaptomus spatulocrenatus	0.30	Persaud et al. 2007	Freshwater	428	41	N	2.5	24	April	2003	Lake Giles		PA, AS, SH, P/US	
Calanoid copepod	Anglaodiaptomus spatulocrenatus	0.28	Persaud et al. 2007	Freshwater	428	41	N	3.9	24	May	2003	Lake Giles		PA, AS, SH, P/US	
Calanoid copepod	Anglaodiaptomus spatulocrenatus	0.29	Persaud et al. 2007	Freshwater	428	41	N	2.5	24	June	2003	Lake Giles		PA, AS, SH, P/US	
Calanoid copepod	Anglaodiaptomus spatulocrenatus	1.24	Persaud et al. 2007	Freshwater	428	41	N	3.2	24	July	2003	Lake Giles		PA, AS, SH, P/US	
Calanoid copepod	Boeckella dialata	2.52	Persaud et al. 2007	Freshwater	1800	45	S	10.4	35	Jan	2004	Alta		MG, SH, PO	
Calanoid copepod	Boeckella dialata	9.01	Persaud et al. 2007	Freshwater	1160	45	S	2.2	1.7	Jan	2004	Burns 1		SH, PO	
Calanoid copepod	Boeckella dialata	5.88	Persaud et al. 2007	Freshwater	1160	45	S	0.39	0.5	Jan	2004	Burns 8		SH, PO, P/US	
Calanoid copepod	Boeckella dialata	2.93	Persaud et al. 2007	Freshwater	1000	44	S	1.5	33	Jan	2004	Hayes		SH, PO	
Calanoid copepod	Boeckella dialata	4.02	Persaud et al. 2007	Freshwater	740	45	S	0.48	10	Jan	2004	Pyramid		MG, PA, SH, PO, P/US	
Calanoid copepod	Boeckella gibbosa	10.60	Persaud et al. 2007	Freshwater	1700	41	S	7.5	12	Jan	1998	Tonček		MG, PA,SH, PO	
Calanoid copepod	Epischura nevadensis	6.51	Persaud et al. 2007	Freshwater	1870	39	N	21	500	Sept	2006	Tahoe		MG, PA, AS, SH, P/US	
Calanoid copepod	Hesperodiaptomus shoshone	0.89	Persaud et al. 2007	Freshwater	3063	44	N	1.3	1	Aug	2003	Rock Pool 3		PA, AS, SH, PO, P/US	
Calanoid copepod	Hesperodiaptomus shoshone	0.78	Persaud et al. 2007	Freshwater	3063	44	N	1.5	1.8	Jul	2001	Rock Pool 4		PA, AS, SH, P/US	
Calanoid copepod	Hesperodiaptomus shoshone	3.76	Persaud et al. 2007	Freshwater	3063	44	N	1.5	1.8	Jul	2003	Rock Pool 4		PA, AS, SH, P/US	
Calanoid copepod	Hesperodiaptomus shoshone	0.30	Persaud et al. 2007	Freshwater	3302	40	N	0.34	0.9	Jul	2002	Elk Roost		SH, PO	
Calanoid copepod	Hesperodiaptomus shoshone	0.92	Persaud et al. 2007	Freshwater	3353	39	N	0.98	1.6	Jul	2002	Forest		SH, PO	
Calanoid copepod	Hesperodiaptomus shoshone	0.04	Persaud et al. 2007	Freshwater	3612	40	N	2.4	5.5	Jul	2002	Green 4		SH	
Calanoid copepod	Leptodiaptomus ashlandi	0.62	Persaud et al. 2007	Freshwater	2713	44	N	0.8	26	July	2003	Lake Beartooth		PA, AS, SH, P/US	
Calanoid copepod	Leptodiaptomus ashlandi	1.46	Persaud et al. 2007	Freshwater	2713	44	N	0.8	26	July	2004	Lake Beartooth		N/A	
Calanoid copepod	Leptodiaptomus minutus	0.20	Persaud et al. 2007	Freshwater	428	41	N	2.5	24	April	2003	Lake Giles		PA, AS, SH, P/US	
Calanoid copepod	Leptodiaptomus minutus	0.15	Persaud et al. 2007	Freshwater	428	41	N	3.9	24	May	2003	Lake Giles		PA, AS, SH	
Calanoid copepod	Leptodiaptomus minutus	0.09	Persaud et al. 2007	Freshwater	428	41	N	2.5	24	June	2003	Lake Giles		PA, AS, SH	
Calanoid copepod	Leptodiaptomus minutus	0.68	Persaud et al. 2007	Freshwater	428	41	N	3.2	24	July	2003	Lake Giles		PA, AS, SH, P/US	
Calanoid copepod	Leptodiaptomus minutus	0.08	Persaud et al. 2007	Freshwater	439	41	N	0.23	13	April	2003	Lake Lacawac		PA, AS, SH, P/US	UVB transparency mean value for all sampling dates
Calanoid copepod	Leptodiaptomus minutus	0.39	Persaud et al. 2007	Freshwater	439	41	N	0.23	13	May	2003	Lake Lacawac		PA, AS, SH, P/US	UVB transparency mean value for all sampling dates
Calanoid copepod	Leptodiaptomus minutus	0.05	Persaud et al. 2007	Freshwater	439	41	N	0.23	13	June	2003	Lake Lacawac		PA, SH, P/US	UVB transparency mean value for all sampling dates
Calanoid copepod	Leptodiaptomus minutus	0.33	Persaud et al. 2007	Freshwater	439	41	N	0.23	13	July	2003	Lake Lacawac		PA, AS, SH, P/US	UVB transparency mean value for all sampling dates
Calanoid copepod	Leptodiaptomus minutus	3.86	Persaud et al. 2007	Freshwater	564	41	N	2.8	4.5	Aug	2006	Lake Earnest		MG, PA, AS, SH, PO, US, P/US	
Calanoid copepod	Leptodiaptomus tyrrelli	2.81	Persaud et al. 2007	Freshwater	3063	44	N	1.5	1.8	Jul	2001	Rock Pool 4		MG, PA, AS, SH, P/US	
Calanoid copepod	Leptodiaptomus tyrrelli	2.01	Persaud et al. 2007	Freshwater	1870	39	N	21	500	Sept	2006	Tahoe		MG, PA, AS, SH, P/US	
Cladocera	Bosmina meridionalis	0.03	Persaud et al. 2007	Freshwater	740	45	S	0.48	10	Jan	2004	Pyramid		N/A	
Cladocera	Ceriodaphnia dubia	0.00	Persaud et al. 2007	Freshwater	1800	45	S	10.4	35	Jan	2004	Alta		N/A	
Cladocera	Ceriodaphnia dubia	0.00	Persaud et al. 2007	Freshwater	1160	45	S	0.39	0.5	Jan	2004	Burns 8		N/A	
Cladocera	Daphnia carinata	0.00	Persaud et al. 2007	Freshwater	1000	44	S	1.5	33	Jan	2004	Hayes		N/A	
Cladocera	Daphnia catawba	0.00	Persaud et al. 2007	Freshwater	428	41	N	2.5	24	April	2003	Lake Giles		N/A	
Cladocera	Daphnia catawba	0.00	Persaud et al. 2007	Freshwater	428	41	N	3.9	24	May	2003	Lake Giles		N/A	
Cladocera	Daphnia catawba	0.02	Persaud et al. 2007	Freshwater	428	41	N	2.5	24	June	2003	Lake Giles		PA, SH, P/US	
Cladocera	Daphnia catawba	0.02	Persaud et al. 2007	Freshwater	428	41	N	3.2	24	July	2003	Lake Giles		PA, AS, SH, P/US	
Cladocera	Daphnia catawba	0.00	Persaud et al. 2007	Freshwater	439	41	N	0.23	13	April	2003	Lake Lacawac		N/A	UVB transparency mean value for all sampling dates
Cladocera	Daphnia catawba	0.00	Persaud et al. 2007	Freshwater	439	41	N	0.23	13	May	2003	Lake Lacawac		N/A	UVB transparency mean value for all sampling dates
Cladocera	Daphnia catawba	0.00	Persaud et al. 2007	Freshwater	439	41	N	0.23	13	June	2003	Lake Lacawac		N/A	UVB transparency mean value for all sampling dates
Cladocera	Daphnia catawba	0.01	Persaud et al. 2007	Freshwater	439	41	N	0.23	13	July	2003	Lake Lacawac		PA, SH	UVB transparency mean value for all sampling dates
Cladocera	Daphnia middendorffiana	0.04	Persaud et al. 2007	Freshwater	3063	44	N	1.5	1.8	Jul	2003	Rock Pool 4		N/A	
Cladocera	Daphnia pulex	0.00	Persaud et al. 2007	Freshwater	3302	40	N	0.34	0.9	Jul	2002	Elk Roost		N/A	
Cladocera	Daphnia pulicaria	0.00	Persaud et al. 2007	Freshwater	2713	44	N	0.8	26	July	2003	Lake Beartooth		N/A	
Cladocera	Daphnia pulicaria	0.05	Persaud et al. 2007	Freshwater	2713	44	N	0.8	26	July	2004	Lake Beartooth		N/A	

Type of MAA Abbreviation	Name
SH	Shinorine
PA	Palythine
PO	Porphyra
AS	Asterina
MY	Mycosporine glycine
US	Usujirene
P	Palythene
MAA332	Unknown
MAA331	Unknown

Cladocera	Daphnia pulicaria	0.05	Persaud et al. 2007	Freshwater	2910	44	N	2.5	45	Jul	2003	Lake Emerald		N/A	
Cladocera	Daphnia pulicaria	0.01	Persaud et al. 2007	Freshwater	<300	58	N	4.7	8	Jul	1996	Bruce Hills		N/A	
Cladocera	Daphnia pulicaria	0.00	Persaud et al. 2007	Freshwater	<300	58	N	1.8	7	Jul	1996	Plateau		N/A	
Cyclopoid copepod	Acanthocyclops vernalis	2.02	Persaud et al. 2007	Freshwater	2910	44	N	2.5	45	Jul	2003	Lake Emerald		PA, AS, SH	
Cyclopoid copepod	Cyclops scutifer	0.16	Persaud et al. 2007	Freshwater	428	41	N	2.5	24	April	2003	Lake Giles		PA, AS, SH, P/US	
Cyclopoid copepod	Cyclops scutifer	0.13	Persaud et al. 2007	Freshwater	428	41	N	3.9	24	May	2003	Lake Giles		PA, AS, SH, P/US	
Cyclopoid copepod	Cyclops scutifer	0.11	Persaud et al. 2007	Freshwater	428	41	N	2.5	24	June	2003	Lake Giles		PA, AS, SH, P/US	
Cyclopoid copepod	Cyclops scutifer	0.25	Persaud et al. 2007	Freshwater	428	41	N	3.2	24	July	2003	Lake Giles		PA, AS, SH, P/US	
Cyclopoid copepod	Cyclops scutifer	0.17	Persaud et al. 2007	Freshwater	439	41	N	0.23	13	April	2003	Lake Lacawac		PA, AS, SH, P/US	UVB transparency mean value for all sampling dates
Cyclopoid copepod	Cyclops scutifer	0.20	Persaud et al. 2007	Freshwater	439	41	N	0.23	13	May	2003	Lake Lacawac		PA, AS, SH, P/US	UVB transparency mean value for all sampling dates
Cyclopoid copepod	Cyclops scutifer	0.16	Persaud et al. 2007	Freshwater	439	41	N	0.23	13	June	2003	Lake Lacawac		PA, AS, SH, P/US	UVB transparency mean value for all sampling dates
Cyclopoid copepod	Cyclops scutifer	0.15	Persaud et al. 2007	Freshwater	439	41	N	0.23	13	July	2003	Lake Lacawac		PA, SH, P/US	UVB transparency mean value for all sampling dates
Cyclopoid copepod	Cyclops scutifer	1.04	Persaud et al. 2007	Freshwater	<300	58	N	4.7	8	Jul	1996	Bruce Hills		PA, AS, PO, P/US	
Cyclopoid copepod	Cyclops scutifer	0.21	Persaud et al. 2007	Freshwater	<300	58	N	1.8	7	Jul	1996	Plateau		PA, AS, PO, P/US	
Cyclopoid copepod	Cyclops scutifer	1.18	Persaud et al. 2007	Freshwater	<300	58	N	7.3	9	Jul	1996	Twin Bruce		PA, AS, PO, P/US	
Cyclopoid copepod	Diacyclops bicuspidatus thomasi	0.86	Persaud et al. 2007	Freshwater	2713	44	N	0.8	26	July	2003	Lake Beartooth		PA, AS, PO, P/US	
Calanoid copepod	Hesperodiaptomus arcticus	0.02	Rautio et al. 2009	Freshwater	N/A	55-56	N	N/A	1	Jul-Aug	2002-2004	Northern Quebec		N/A	Only one sample
Calanoid copepod	Leptodiaptomus minutus	5.10	Rautio et al. 2009	Freshwater	N/A	55-56	N	N/A	1	Jul-Aug	2002-2004	Northern Quebec		N/A	
Cladocera	Daphnia middendorffiana	1.00	Rautio et al. 2009	Freshwater	N/A	55-81	N	N/A	N/A	Jul-Aug	2002-2004	Northern Canada		N/A	Only one sample
Calanoid copepod	Arctodiaptomus jurisowitchi	1.71	Sommaruga 2010	Freshwater	4890	27	N	9.37	0.6	Oct	2014	Lake 14		PO	Sometimes a small peak with SH
Calanoid copepod	Arctodiaptomus jurisowitchi	0.56	Sommaruga 2010	Freshwater	5160	27	N	12.8	4.8	Oct	2014	Lake 15		PO	Sometimes a small peak with SH
Calanoid copepod	Arctodiaptomus jurisowitchi	0.03	Sommaruga 2010	Freshwater	5067	27	N	7.8	14.8	Oct	2014	Lake 10		PO	Sometimes a small peak with SH
Calanoid copepod	Arctodiaptomus jurisowitchi	0.03	Sommaruga 2010	Freshwater	5213	27	N	12.5	8.2	Oct	2014	Lake 9		PO	Sometimes a small peak with SH
Cyclopoid copepod	Cyclops abyssorum tatricus	2.20	Tartarotti and Sommaruga 2006	Freshwater	2417	47	N	N/A	9.9	May	2001	Gossenköllesee	Female	SH, AS, PA	Data also available on other life stages of copepods. These three MAAs were always present. Other MAAs occured in lower concentrations.
Cyclopoid copepod	Cyclops abyssorum tatricus	8.03	Tartarotti and Sommaruga 2006	Freshwater	2417	47	N	N/A	9.9	Jul	2001	Gossenköllesee	Female	SH, AS, PA	Data also available on other life stages of copepods. These three MAAs were always present. Other MAAs occured in lower concentrations.
Cyclopoid copepod	Cyclops abyssorum tatricus	5.60	Tartarotti and Sommaruga 2006	Freshwater	2417	47	N	N/A	9.9	Jul	2001	Gossenköllesee	Female	SH, AS, PA	Data also available on other life stages of copepods. These three MAAs were always present. Other MAAs occured in lower concentrations.
Cyclopoid copepod	Cyclops abyssorum tatricus	7.03	Tartarotti and Sommaruga 2006	Freshwater	2417	47	N	N/A	9.9	Aug	2001	Gossenköllesee	Female	SH, AS, PA	Data also available on other life stages of copepods. These three MAAs were always present. Other MAAs occured in lower concentrations.
Cyclopoid copepod	Cyclops abyssorum tatricus	7.01	Tartarotti and Sommaruga 2006	Freshwater	2417	47	N	N/A	9.9	Aug	2001	Gossenköllesee	Female	SH, AS, PA	Data also available on other life stages of copepods. These three MAAs were always present. Other MAAs occured in lower concentrations.
Cyclopoid copepod	Cyclops abyssorum tatricus	5.48	Tartarotti and Sommaruga 2006	Freshwater	2417	47	N	N/A	9.9	Nov	2001	Gossenköllesee	Female	SH, AS, PA	Data also available on other life stages of copepods. These three MAAs were always present. Other MAAs occured in lower concentrations.
Cyclopoid copepod	Cyclops abyssorum tatricus	3.90	Tartarotti and Sommaruga 2006	Freshwater	2417	47	N	N/A	9.9	Jan	2002	Gossenköllesee	Female	SH, AS, PA	Data also available on other life stages of copepods. These three MAAs were always present. Other MAAs occured in lower concentrations.
Cyclopoid copepod	Cyclops abyssorum tatricus	2.90	Tartarotti and Sommaruga 2006	Freshwater	2417	47	N	N/A	9.9	Jan	2002	Gossenköllesee	Female	SH, AS, PA	Data also available on other life stages of copepods. These three MAAs were always present. Other MAAs occured in lower concentrations.
Cyclopoid copepod	Cyclops abyssorum tatricus	3.22	Tartarotti and Sommaruga 2006	Freshwater	2417	47	N	N/A	9.9	Mar	2002	Gossenköllesee	Female	SH, AS, PA	Data also available on other life stages of copepods. These three MAAs were always present. Other MAAs occured in lower concentrations.
Cyclopoid copepod	Cyclops abyssorum tatricus	2.78	Tartarotti and Sommaruga 2006	Freshwater	2417	47	N	N/A	9.9	Apr	2002	Gossenköllesee	Female	SH, AS, PA	Data also available on other life stages of copepods. These three MAAs were always present. Other MAAs occured in lower concentrations.
Cyclopoid copepod	Cyclops abyssorum tatricus	4.23	Tartarotti and Sommaruga 2006	Freshwater	2417	47	N	N/A	9.9	Jun	2002	Gossenköllesee	Female	SH, AS, PA	Data also available on other life stages of copepods. These three MAAs were always present. Other MAAs occured in lower concentrations.
Cyclopoid copepod	Cyclops abyssorum tatricus	9.23	Tartarotti and Sommaruga 2006	Freshwater	2417	47	N	N/A	9.9	Jun	2002	Gossenköllesee	Female	SH, AS, PA	Data also available on other life stages of copepods. These three MAAs were always present. Other MAAs occured in lower concentrations.
Cyclopoid copepod	Cyclops abyssorum tatricus	7.37	Tartarotti and Sommaruga 2006	Freshwater	2417	47	N	N/A	9.9	Jul	2002	Gossenköllesee	Female	SH, AS, PA	Data also available on other life stages of copepods. These three MAAs were always present. Other MAAs occured in lower concentrations.
Cyclopoid copepod	Cyclops abyssorum tatricus	1.66	Tartarotti and Sommaruga 2006	Freshwater	2417	47	N	N/A	9.9	May	2001	Gossenköllesee	Male	SH, AS, PA	Data also available on other life stages of copepods. These three MAAs were always present. Other MAAs occured in lower concentrations.
Cyclopoid copepod	Cyclops abyssorum tatricus	6.51	Tartarotti and Sommaruga 2006	Freshwater	2417	47	N	N/A	9.9	Jul	2001	Gossenköllesee	Male	SH, AS, PA	Data also available on other life stages of copepods. These three MAAs were always present. Other MAAs occured in lower concentrations.
Cyclopoid copepod	Cyclops abyssorum tatricus	3.59	Tartarotti and Sommaruga 2006	Freshwater	2417	47	N	N/A	9.9	Jul	2001	Gossenköllesee	Male	SH, AS, PA	Data also available on other life stages of copepods. These three MAAs were always present. Other MAAs occured in lower concentrations.
Cyclopoid copepod	Cyclops abyssorum tatricus	4.67	Tartarotti and Sommaruga 2006	Freshwater	2417	47	N	N/A	9.9	Jul	2001	Gossenköllesee	Male	SH, AS, PA	Data also available on other life stages of copepods. These three MAAs were always present. Other MAAs occured in lower concentrations.
Cyclopoid copepod	Cyclops abyssorum tatricus	6.10	Tartarotti and Sommaruga 2006	Freshwater	2417	47	N	N/A	9.9	Aug	2001	Gossenköllesee	Male	SH, AS, PA	Data also available on other life stages of copepods. These three MAAs were always present. Other MAAs occured in lower concentrations.
Cyclopoid copepod	Cyclops abyssorum tatricus	5.02	Tartarotti and Sommaruga 2006	Freshwater	2417	47	N	N/A	9.9	Aug	2001	Gossenköllesee	Male	SH, AS, PA	Data also available on other life stages of copepods. These three MAAs were always present. Other MAAs occured in lower concentrations.
Cyclopoid copepod	Cyclops abyssorum tatricus	6.70	Tartarotti and Sommaruga 2006	Freshwater	2417	47	N	N/A	9.9	Oct	2001	Gossenköllesee	Male	SH, AS, PA	Data also available on other life stages of copepods. These three MAAs were always present. Other MAAs occured in lower concentrations.
Cyclopoid copepod	Cyclops abyssorum tatricus	3.96	Tartarotti and Sommaruga 2006	Freshwater	2417	47	N	N/A	9.9	Nov	2001	Gossenköllesee	Male	SH, AS, PA	Data also available on other life stages of copepods. These three MAAs were always present. Other MAAs occured in lower concentrations.
Cyclopoid copepod	Cyclops abyssorum tatricus	3.42	Tartarotti and Sommaruga 2006	Freshwater	2417	47	N	N/A	9.9	Dec	2001	Gossenköllesee	Male	SH, AS, PA	Data also available on other life stages of copepods. These three MAAs were always present. Other MAAs occured in lower concentrations.
Cyclopoid copepod	Cyclops abyssorum tatricus	2.39	Tartarotti and Sommaruga 2006	Freshwater	2417	47	N	N/A	9.9	Jan	2002	Gossenköllesee	Male	SH, AS, PA	Data also available on other life stages of copepods. These three MAAs were always present. Other MAAs occured in lower concentrations.
Cyclopoid copepod	Cyclops abyssorum tatricus	2.01	Tartarotti and Sommaruga 2006	Freshwater	2417	47	N	N/A	9.9	Jan	2002	Gossenköllesee	Male	SH, AS, PA	Data also available on other life stages of copepods. These three MAAs were always present. Other MAAs occured in lower concentrations.
Cyclopoid copepod	Cyclops abyssorum tatricus	1.70	Tartarotti and Sommaruga 2006	Freshwater	2417	47	N	N/A	9.9	Feb	2002	Gossenköllesee	Male	SH, AS, PA	Data also available on other life stages of copepods. These three MAAs were always present. Other MAAs occured in lower concentrations.
Cyclopoid copepod	Cyclops abyssorum tatricus	2.22	Tartarotti and Sommaruga 2006	Freshwater	2417	47	N	N/A	9.9	Mar	2002	Gossenköllesee	Male	SH, AS, PA	Data also available on other life stages of copepods. These three MAAs were always present. Other MAAs occured in lower concentrations.
Cyclopoid copepod	Cyclops abyssorum tatricus	1.74	Tartarotti and Sommaruga 2006	Freshwater	2417	47	N	N/A	9.9	Apr	2002	Gossenköllesee	Male	SH, AS, PA	Data also available on other life stages of copepods. These three MAAs were always present. Other MAAs occured in lower concentrations.
Cyclopoid copepod	Cyclops abyssorum tatricus	1.51	Tartarotti and Sommaruga 2006	Freshwater	2417	47	N	N/A	9.9	Apr	2002	Gossenköllesee	Male	SH, AS, PA	Data also available on other life stages of copepods. These three MAAs were always present. Other MAAs occured in lower concentrations.
Cyclopoid copepod	Cyclops abyssorum tatricus	1.47	Tartarotti and Sommaruga 2006	Freshwater	2417	47	N	N/A	9.9	May	2002	Gossenköllesee	Male	SH, AS, PA	Data also available on other life stages of copepods. These three MAAs were always present. Other MAAs occured in lower concentrations.
Cyclopoid copepod	Cyclops abyssorum tatricus	2.36	Tartarotti and Sommaruga 2006	Freshwater	2417	47	N	N/A	9.9	Jun	2002	Gossenköllesee	Male	SH, AS, PA	Data also available on other life stages of copepods. These three MAAs were always present. Other MAAs occured in lower concentrations.
Cyclopoid copepod	Cyclops abyssorum tatricus	4.09	Tartarotti and Sommaruga 2006	Freshwater	2417	47	N	N/A	9.9	Jun	2002	Gossenköllesee	Male	SH, AS, PA	Data also available on other life stages of copepods. These three MAAs were always present. Other MAAs occured in lower concentrations.
Cyclopoid copepod	Cyclops abyssorum tatricus	4.85	Tartarotti and Sommaruga 2006	Freshwater	2417	47	N	N/A	9.9	Jul	2002	Gossenköllesee	Male	SH, AS, PA	Data also available on other life stages of copepods. These three MAAs were always present. Other MAAs occured in lower concentrations.
Calanoid copepod	Boeckella gibbosa	8.64	Tartarotti et al. 2004	Freshwater	1950	41	S	N/A	5	Dec	1997	L. Schmoll		PO, MY, SH, MAA331, PA, PO, AS, P	
Calanoid copepod	Boeckella gibbosa	7.80	Tartarotti et al. 2004	Freshwater	1750	41	S	N/A	0.3	Dec	1997	Pond		PO, MY, SH, MAA331, PA, US	
Calanoid copepod	Boeckella gibbosa	6.94	Tartarotti et al. 2004	Freshwater	1650	41	S	N/A	8	Dec	1997	L. Tempanos		PO, MY, SH, MAA331, PA, AS, P	
Calanoid copepod	Boeckella gibbosa	6.35	Tartarotti et al. 2004	Freshwater	1700	41	S	N/A	12	Dec	1997	L. Toncek		PO, MY, SH, MAA331, US	
Calanoid copepod	Boeckella gibbosa	5.41	Tartarotti et al. 2004	Freshwater	1100	41	S	N/A	10	Dec	1997	L. Negra		PO, MY, SH, MAA331, PA, AS, P	
Calanoid copepod	Boeckella gibbosa	3.79	Tartarotti et al. 2004	Freshwater	1550	41	S	N/A	25	Dec	1997	L. Jakob		PO, MY, SH, MAA331, PA	Also small amounts of other MAAs
Calanoid copepod	Boeckella gracilipes	2.63	Tartarotti et al. 2004	Freshwater	705	40	S	N/A	25-80	Dec	1997	Alicura		PO, MY, SH, MAA331, PA, US	
Calanoid copepod	Boeckella gracilipes	2.24	Tartarotti et al. 2004	Freshwater	1545	41	S	N/A	5	Dec	1997	L. Verde		PO, MY, MAA331	Also small amounts of other MAAs
Calanoid copepod	Boeckella gracilipes	0.19	Tartarotti et al. 2004	Freshwater	764	41	S	N/A	12	Dec	1997	L. Trebol		PO	Also small amounts of other MAAs
Calanoid copepod	Boeckella meteoris	2.39	Tartarotti et al. 2004	Freshwater	590	40	S	N/A	~1	Dec	1997	L. Puesto		PO, MY, SH	Also small amounts of other MAAs
Calanoid copepod	Parabroteas sarsi	1.49	Tartarotti et al. 2004	Freshwater	590	40	S	N/A	~1	Dec	1997	L. Toro		PO, MY, SH	Also small amounts of other MAAs
Calanoid copepod	Parabroteas sarsi	1.31	Tartarotti et al. 2004	Freshwater	590	40	S	N/A	~1	Dec	1997	L. Puesto		PO, MY, SH	Also small amounts of other MAAs
Calanoid copepod	Parabroteas sarsi	1.27	Tartarotti et al. 2004	Freshwater	590	40	S	N/A	~1	Dec	1997	L. Flamingo		PO, MY, SH	Also small amounts of other MAAs
Cyclopoid copepod	Cyclops abyssorum tatricus	11.08	Tartarotti et al. 2014	Freshwater	2416	47	N	21.27	15	Jul	2011	Faselfadsee4		SH dominant	
Cyclopoid copepod	Cyclops abyssorum tatricus	5.24	Tartarotti et al. 2014	Freshwater	2483	47	N	5.62	8.1	Sept	2011	Mutterbergersee		SH dominant	
Cyclopoid copepod	Cyclops abyssorum tatricus	2.98	Tartarotti et al. 2014	Freshwater	2414	47	N	1.19	17	Aug	2011	Faselfadsee3		SH dominant	
Cyclopoid copepod	Cyclops abyssorum tatricus	8.94	Tartarotti et al. 2017	Freshwater	2416	47	N	24.4	15	Jul	2010	Faselfadsee4	CV-adults	SH, PO, PA, AS, US, P	
Cyclopoid copepod	Cyclops abyssorum tatricus	8.40	Tartarotti et al. 2017	Freshwater	2416	47	N	21.3	15	Jul	2011	Faselfadsee4	CV-adults	SH, PO, PA, AS, US, P	
Cyclopoid copepod	Cyclops abyssorum tatricus	10.38	Tartarotti et al. 2017	Freshwater	2416	47	N	16	15	Aug	2011	Faselfadsee4	CV-adults	SH, PO, PA, AS, US, P	
Cyclopoid copepod	Cyclops abyssorum tatricus	2.10	Tartarotti et al. 2017	Freshwater	2425	47	N	3.2	6.3	Jul-Aug	2010-2011	Weißsee	CV-adults	SH, PO, AS	
Cyclopoid copepod	Cyclops abyssorum tatricus	0.40	Tartarotti et al. 2017	Freshwater	2263	47	N	2.4	10	Jul-Aug	2010-2011	Faselfadsee6	CV-adults	SH, PO,	
Cyclopoid copepod	Cyclops abyssorum tatricus	1.76	Tartarotti et al. 2017	Freshwater	2420	47	N	2.7	17	Jul	2010	Faselfadsee3	CV-adults	SH, PO, PA, AS, US, P	
Cyclopoid copepod	Cyclops abyssorum tatricus	2.78	Tartarotti et al. 2017	Freshwater	2420	47	N	3.1	17	Jul	2011	Faselfadsee3	CV-adults	SH, PO, PA, AS, US, P	
Cyclopoid copepod	Cyclops abyssorum tatricus	1.99	Tartarotti et al. 2017	Freshwater	2420	47	N	1.2	17	Aug	2011	Faselfadsee3	CV-adults	SH, PO, PA, AS, US, P	
Cyclopoid copepod	Cyclops abyssorum tatricus	1.48	Tartarotti et al. 2017	Freshwater	2234	47	N	0.5	24	Jul-Aug	2010-2011	Rifflsee	CV-adults	SH, PO, PA, AS, US, P	
Cyclopoid copepod	Cyclops abyssorum tatricus	9.16	Tartatortti et al. 2018	Freshwater	2413	47	N	N/A	9.9	Aug	2013	Gossenköllesee		N/A	
Cyclopoid copepod	Cyclops abyssorum tatricus	13.01	Tartatortti et al. 2018	Freshwater	2413	47	N	N/A	9.9	Oct	2013	Gossenköllesee		N/A	
Cyclopoid copepod	Cyclops abyssorum tatricus	6.28	Tartatortti et al. 2018	Freshwater	2413	47	N	N/A	9.9	Dec	2013	Gossenköllesee		N/A	
Cyclopoid copepod	Cyclops abyssorum tatricus	3.96	Tartatortti et al. 2018	Freshwater	2413	47	N	N/A	9.9	Mar	2014	Gossenköllesee		N/A	
Cyclopoid copepod	Cyclops abyssorum tatricus	4.68	Tartatortti et al. 2018	Freshwater	2413	47	N	N/A	9.9	Apr	2014	Gossenköllesee		N/A	
Cyclopoid copepod	Cyclops abyssorum tatricus	7.72	Tartatortti et al. 2018	Freshwater	2413	47	N	N/A	9.9	Jul	2014	Gossenköllesee		N/A	
